# Idiopathic membranous nephropathy in a patient diagnosed with IgG4-related disease: A case report

**DOI:** 10.1097/MD.0000000000022817

**Published:** 2020-10-16

**Authors:** XiaoYing Ma, HaiPing Xu, Jing Yi Sun, Yuresha Surangani Siyabalagaba Gedara, FuYun Sun

**Affiliations:** Nephrology Department, Cangzhou Central Hospital, Yunhe Qu, Cangzhou, Hebei, China.

**Keywords:** case report, idiopathic membranous nephropathy, immunoglobulin G4-related disease, membranous nephropathy associated with immunoglobulin G4-related disease, monoclonal immunoglobulinemia syndrome

## Abstract

**Rationale:**

Immunoglobulin (Ig) G4-related disease (IgG4-RD) is a newly recognized, systemic disease. Membranous nephropathy is the most common glomerular lesion in IgG4- related kidney disease. However, the lack of relationship with IgG4-related kidney disease and monoclonal gammopathy of undetermined significance (MGUS) warrants investigation of the potential mechanisms.

**Patient concerns:**

A 62-year-old patient was diagnosed with IgG4-RD, tubulointerstitial nephritis, retroperitoneal fibrosis. After 2 years, she was presented with proteinuria, hypoproteinemia, facial, and bilateral lower limb edema. Furthermore, this patient exhibited deposits of IgG k of monoclonal hyperplasia, and bone marrow plasma cell count was 2.5%.

**Diagnosis:**

The patient was diagnosed with nephrotic syndrome, acute kidney injury, and MGUS. The pathological diagnosis was IgG4-related tubulointerstitial nephritis, IgG4-related membranous nephropathy.

**Interventions:**

The patient was treated with intravenous methylprednisolone (40 mg daily), which was changed to oral prednisone 50 mg/d after 2 months.

**Outcomes:**

After 1 month, the patient exhibited a rapid response only with corticosteroid, and experienced partial remission of serum albumin and proteinuria.

**Lessons:**

This case may suggest a possible relationship between IgG4-RD and MGUS, provide some guidance for investigating the mechanism between them.

## 1 Introduction

Immunoglobulin (Ig) G4-related disease (IgG4-RD) is defined as a multiorgan systemic disorder with pathological findings affecting a wide range of organ systems. Immunoglobulin G4-related disease is a recently identified clinical entity characterized by increased serum IgG4 levels and infiltration of IgG4-positive cells into various organs.^[1–6]^ IgG4-related tubulointerstitial nephritis (TIN) has been described in the kidney recently,^[7,8]^ glomerular disease has also been reported in a few case reports and noted incidentally in some series investigating IgG4-related TIN.^[9,10]^ We report a case involving a patient with IgG4-RD who developed nephrotic syndrome and membranous nephropathy (MN).

## 2 Case presentation

The present study was approved by the Ethics Committee of the Cangzhou Central Hospital, located in Cangzhou City, Hebei Province, China. Informed consent for publication of anonymized case details was obtained from the patient. In June 2015, a 62-year-old woman was referred to a local hospital for the first time with a 1-month history of fatigue and anorexia. Her urinalysis was normal, and renal function parameter values were serum creatinine (sCr), 899 μmol/L, blood urea nitrogen, 20.57 mmol/L. Abdominopelvic computed tomography revealed bilateral hydronephrosis, ureterectasia, and a normal size kidney. She was diagnosed with obstructive nephropathy and acute kidney disease. Double-J stents were inserted into the bilateral ureters, after which sCr level dropped from 899 μmol/L to 366 μmol/L. Ten days before admission, she was diagnosed with “urinary tract infection”. Her weight was 63 kg, blood pressure was 165/85 mm Hg and, except for a palpable right cervical lymph node, the remainder of the physical examination was unremarkable. Lymph node biopsy revealed chronic lymphadenitis and reactive lymphoid proliferations. Immunohistochemical analysis revealed IgG4 and IgG + plasma cells < 40%, IgG4-positive plasma cells < 10 cells/high-power field (HPF). Ultrasound of the kidney yielded normal findings. Abdominal computed tomography revealed retroperitoneal fibrosis. Results of laboratory investigations are summarized in Table 1.

**Table 1:**
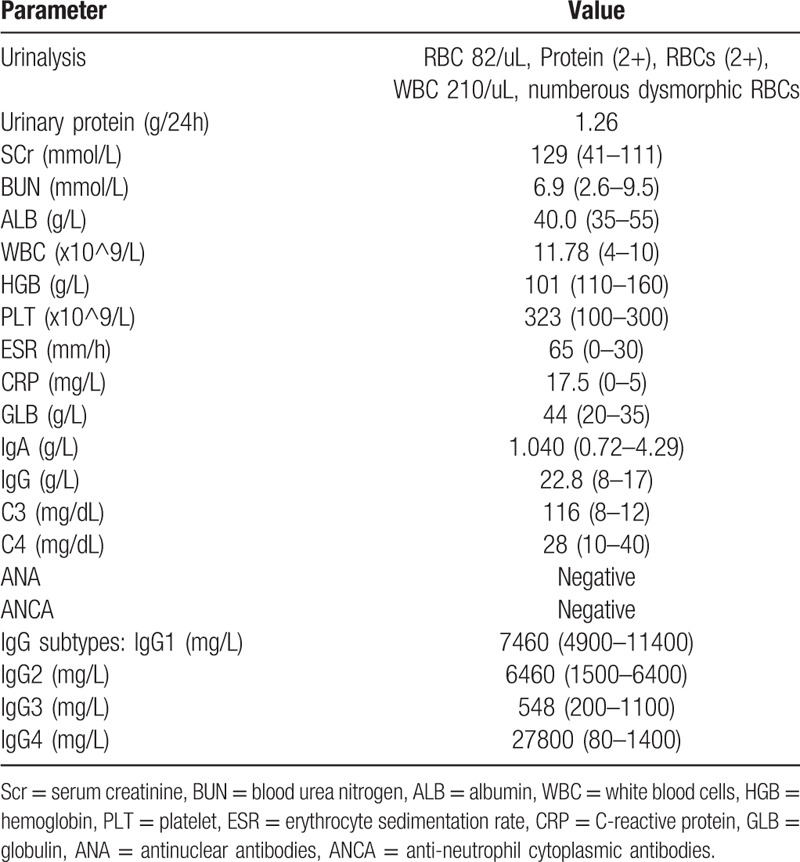
Laboratory data.

Because this patient had a high erythrocyte sedimentation rate and high levels of IgG and C-reactive protein, plasma cell disease was plausible. However, bone marrow aspiration revealed mature plasma cells (2.5%), and electrophoresis and immunofixation of 24 hour urine collection was normal; as such, evidence supporting the diagnosis was insufficient. The clinical presentation, together with retroperitoneal fibrosis, palpable right cervical lymph node, and markedly increased serum IgG4 levels led to a diagnosis of IgG4-RD. The patient was treated with antibiotics before oral prednisolone 30 mg daily because she had a history of urinary tract infection. Two months later, laboratory results were as follows: globulin (GLB) 37 g/L; sCr, 94 μmol/L; IgG 15.53 mmol/L; and 24 hour urinary protein, 0.74 g proteinuria/d (urine volume 2.55 L). Prednisolone regulation decreased until drug withdrawal, and uridine triphosphate level returned to normal. The patient felt well and, therefore, was not followed-up for 2 years.

In March 2017, she was referred to the authors’ department for the second time with edema of the lower limbs. Except for facial and bilateral lower limb edema, the remainder of the physical examination was unremarkable. Ultrasound of the kidney was normal and computed tomography of the abdomen revealed no retroperitoneal fibrosis. Other laboratory results are summarized in Table 2.

**Table 2:**
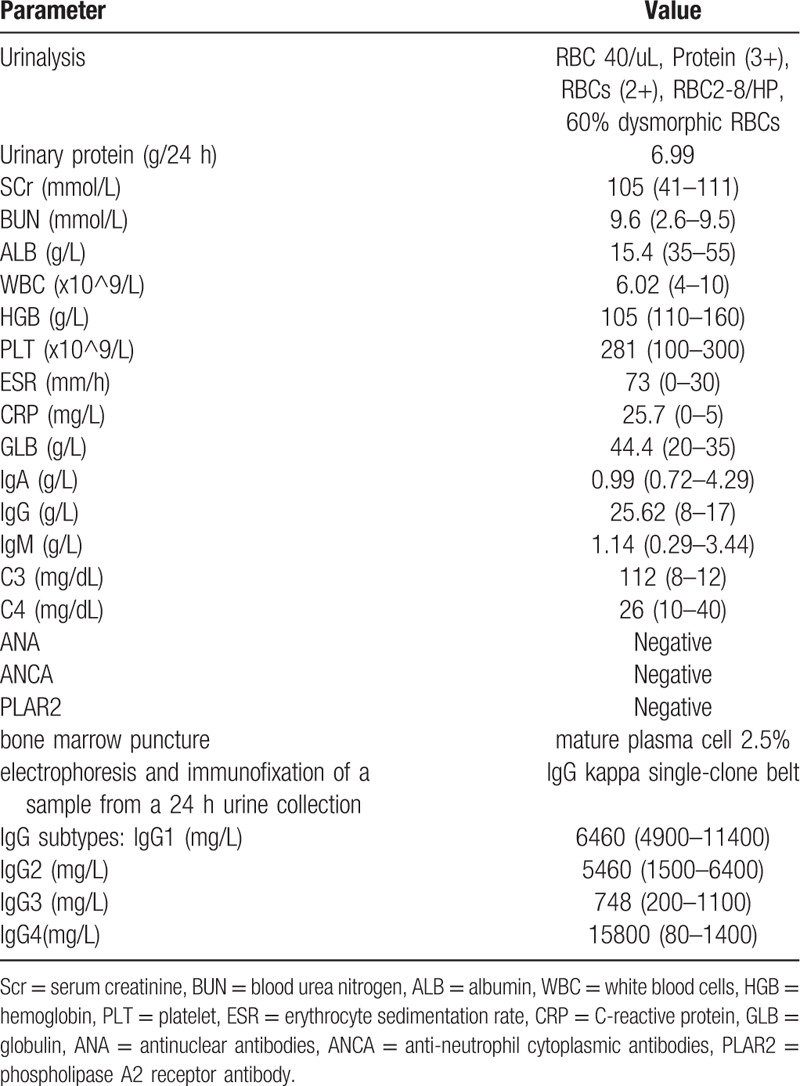
Laboratory findings.

**Figure 1:**
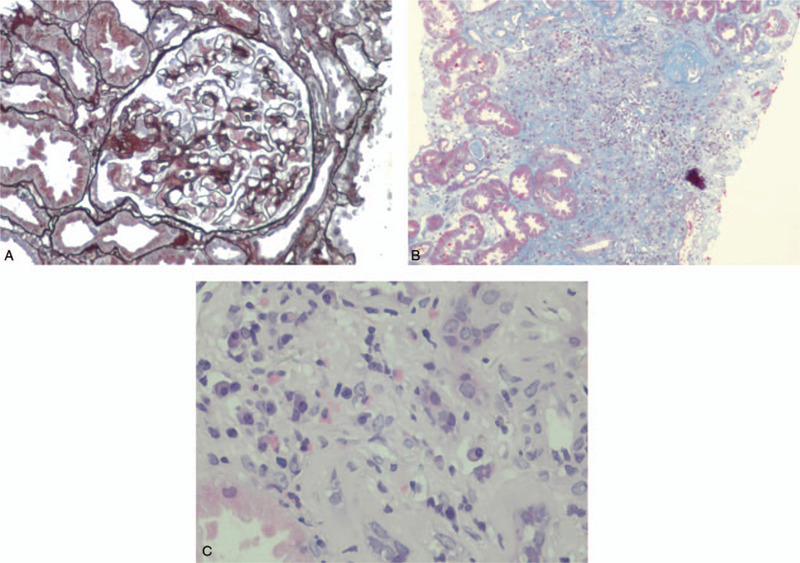
Light microscopy findings from renal biopsy. (A) Glomeruli exhibiting segmental spike formation and slightly thickened glomerular basement membranes (PAM trichrome stain, original magnification ×200). (B) Interstitium exhibiting cell infiltration and interstitial fibrosis with tubule atrophy (Masson trichrome stain, original magnification ×100). (C) Details of the inflammatory infiltrate, with numerous plasma cells and other mononuclear cells (hematoxylin and eosin stain, original magnification ×400).

**Figure 2:**
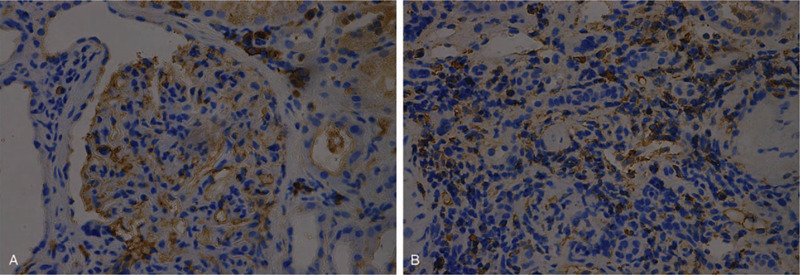
(A) Immunoglobulin G4 diffusing into the glomerular capillary walls. (B) immunoglobulinG4 staining was also observed in the infiltrated plasma cells in the renal interstitium (original magnification ×400).

**Figure 3:**
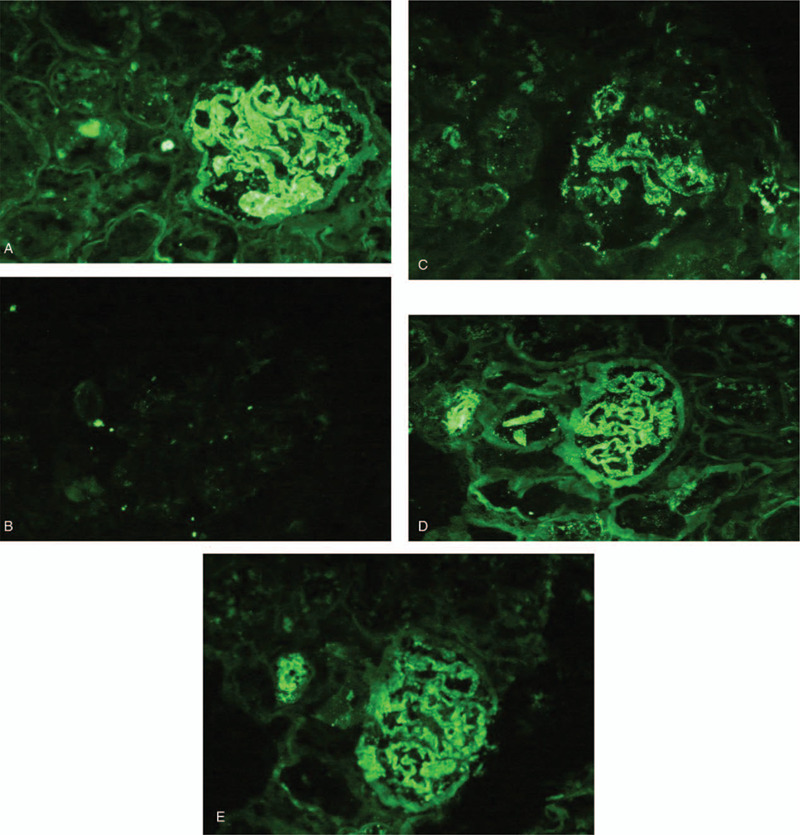
Immunoglobulin (Ig) G, IgM, C3, FRA, κ light chains, λ light chains were granular deposited along the glomerular capillary walls. (A) IgG (original magnification ×400); (B) IgM (original magnification ×400); (C) C3 (original magnification ×400); (D) k (original magnification ×400); (E) λ (original magnification ×400).

Due to suspected autoimmune disease, renal biopsy was performed. The specimen assessed under light microscopy contained 17 glomeruli, of which 5 were globally sclerotic, while 1 glomerulus exhibited cell fibrotic crescent formation. Non-sclerosed glomeruli exhibited a minimal increase in the mesangial matrix. Glomerular basement membranes were slightly thickened. Fine subepithelial deposits on the glomerular capillary walls were noticed on segmental glomerular basement membranes on PAM-Masson trichrome staining (Fig. 1A). Interstitial cell infiltration was observed in focal areas of the renal interstitium. Infiltration consisted of mononuclear cells and plasma cells. Fibrosis was also evident in the inflamed areas (Fig. 1B, 1C). Immunohistochemistry results revealed strong IgG4 staining of the infiltrated plasma cells in the renal interstitium. IgG4 also stained positive on the glomerular capillary walls (Fig. 2A, 2B). Routine immunofluorescence revealed IgG (+++), IgM (+), C3 (++), FRA (+), and κ light chains (++) λ light chains (++) granular deposits along the glomerular capillary walls. Glomeruli were negative for IgA and C1q (Fig. 3A-3E).

**Figure 4:**
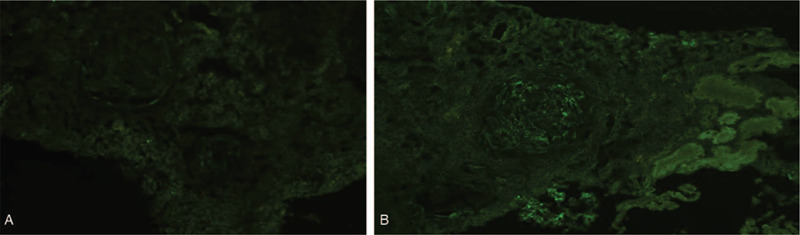
Diffuse weak immunoglobulin (Ig) G1 and strong IgG4 deposition in the glomeruli and the interstitium. (A) IgG1 (original magnification ×400); (B) IgG4 (original magnification ×400).

**Figure 5:**
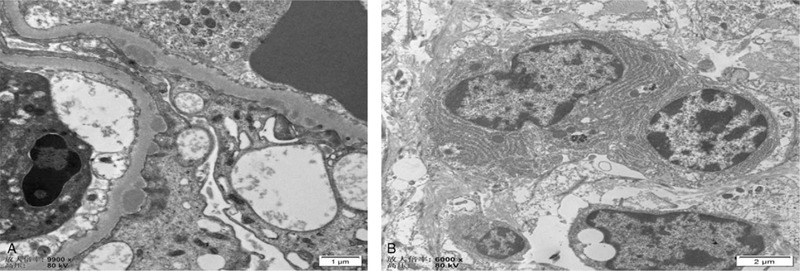
Electron microscopy revealing scattered subepithelial deposits (A). Plasma cells in the renal interstitium (B).

Immunofluorescence staining for IgG4 subclasses revealed diffuse and global deposits of IgG1 and IgG4 along the glomerular capillary walls and interstitium (Fig. 4A, 4B). Electron microscopy revealed small subepithelial electron-dense deposition and focal effacement of podocyte foot processes. The mesangium did not contain electron-dense deposits (Fig. 5A, 5B). The patient was diagnosed with nephrotic syndrome, acute kidney injury, and monoclonal-gammopathy of undetermined significance (MGUS). Based on pathology results, the patient was diagnosed with IgG4-related TIN, IgG4-related MN. She was started on intravenous methylprednisolone at a dose of 40 mg daily, which was then changed to prednisone 50 mg/d per os after 2 months. One month later, laboratory data investigations revealed the following: albumin (ALB) 28 g/L; GLB 26.1 g/L; sCr 81 μmol/L; and blood urea nitrogen, 9.9 mmol/L. Two months later, laboratory investigations revealed the following: uridine triphosphate, 4.0 g (2.0 L); ALB, 31.8 g/L; GLB, 26.9 g/L; IgG, 11.22 g/L; IgG4,1340 mg/L, and SCr, 75.0 μmol/L.

## 3 Discussion

In this case, we reported a patient diagnosed with IgG4-RD who developed MN and MGUS. The specific findings speculated an association between IgG4-RD and MGUS.

IgG4-RD is a recently recognized, distinct, systemic disease with unique histopathological features. Initial reports describing extra-pancreatic manifestations associated with autoimmune pancreatitis appeared in the literature have improved the understanding and recognition of this distinctive systemic disease.^[11]^ The concept of IgG4-RD was recently recognized by Kamisawa et al in 2003.^[12]^ The name and definition of IgG4-RD were established in 2010.^[13]^ Criteria used to diagnose IgG4 disease are as follows: clinically, diffuse/localized enlargement, or mass formation in 1 or more organs, and high serum IgG4 level (> 135 mg/dL); and, pathologically, > 10 IgG4^+^cells/HPF and IgG4^+^/IgG cell ratio > 40%.^[14]^ IgG4-RD has been observed in many organ systems, including the pancreas and other gastrointestinal sites, salivary or lachrymal glands, lung, breast, lymph node, and retroperitoneum.^[1,15,16]^ It is characterized by high serum IgG4 levels and IgG4-positive cell-dense infiltrate.^[17]^

IgG4- related kidney disease (IgG4-RKD) is a comprehensive term for renal lesions associated with IgG4-RD.^[18]^ A small case series reported that 28 of 34 (83%) IgG4-RD patients had IgG4-RKD.^[19,20]^ However, it has been noted that Japanese cases differ from those in the West. Approximately 98% of IgG4-RD patients had kidney disease.^[21]^ In the kidney, IgG4-RD manifests most commonly as TIN. IgG4-related TIN is diagnosed based on a combination of clinical, serological, and radiological findings, along with pathological features.^[16]^ Patients with IgG4-TIN are usually ≥ 65 years of age, of which the majority (80%) are male.^[17,20]^ Patients with IgG4-RKD often exhibit lesions in other organs, although others may exhibit involvement in only a single organ.^[22]^ Almost 80% of IgG4-TIN patients have elevated levels of serum total IgG or IgG4.^[10]^ Under light microscopy, IgG4-TIN exhibits a plasma cell-rich interstitial inflammatory cell infiltrate. In addition, characteristic fibrosis is considered to be an important diagnostic finding. Fibrosis exhibits a “storiform” pattern, as seen in the kidney.^[20]^ Immunostaining for IgG4 plasma cells is helpful in distinguishing IgG4-TIN. Dense lymphoplasmacytic infiltrate with 10 IgG4 plasma cells/HPF and/or IgG4/IgG plasma cell ratio ≥ 40%.^[20]^ Recent publications from Japan and North America have proposed diagnostic criteria for IgG4-TIN.^[8,20]^ Our patient was an elderly woman with retroperitoreal fibrosis, a palpable right cervical lymph node, high levels of serum IgG, IgG4, and acute renal injury, with typical features of kidney biopsy. Considering these factors, we confirmed the diagnosis of IgG4-related TIN.

MN is the most common glomerular lesion in IgG4-RKD and, among them, 7% to 10% of patients have IgG4-TIN.^[23,24]^ Using immunofluorescence, IgG, and C3 global staining in GBM, immunofluorescence staining for IgG subclasses revealed that IgG4 was deposited in GBM.^[25]^ Immunostaining for phospholipase A2 receptor is negative.^[26]^ IgG4-related MN should be suspected in IgG4-RD patients with significant proteinuria, and those with MN on renal biopsy, and an appropriate clinical history should be evaluated for IgG4-RD. Our patient presented with significant proteinuria and hematuria during the second hospitalization. This suggested the presence of glomerular disease in addition to IgG4-related TIN. Kidney biopsy revealed MN. Medical history was significant for retroperitoneal fibrosis, and a palpable right cervical lymph node was also likely due to IgG4-RD. Moreover, circulating anti-PLA2R antibodies were negative, which suggested that MN was IgG4-related membranous glomerulonephritis.

In addition, the patient exhibited deposits of IgG k of monoclonal hyperplasia, and bone marrow plasma cell count was 2.5%. MGUS is characterized by the presence of a monoclonal gammopathy without end organ damage.^[27]^ MGUS requires serum monoclonal protein and bone marrow plasma cell levels to be < 3 g/dL and 10%, respectively. Accordingly, our patient could be diagnosed with MGUS. Meanwhile, monoclonal gammopathy of renal significance should be suspected when the monoclonal protein plays a direct role in kidney disease. Monoclonal protein studies should be performed to match the monoclonal protein in circulation with monoclonal deposits in the kidney. Treatment of monoclonal gammopathy of renal significance-related kidney diseases should be tailored to the clone responsible to protect the organs. A kidney biopsy of our patient revealed that κ light and λ light chains were deposited along the glomerular capillary walls, and she exhibited a sensitive response to hormone. Therefore, it was ruled out that monoclonal protein deposited in the kidney caused IgG4-RD. However, it remains unclear whether IgG4-RD could cause MGUS. One study reported that patients with IgG4-RD with or without renal insufficiency had increased serum free light chain levels.^[28]^ Another study showed that approximately 1-half (43%) of the IgG4-RD patients exhibited an increased and abnormal κ:λ ratio.^[29]^ Usually, an abnormal ratio is associated in the context of monoclonal gammopathy.^[30]^ We found no reports that IgG4-RD could cause MGUS. According to the results of our study, there is a possible relationship between IgG4-RD and MGUS, which could be due to an unknown underlying mechanism(s) of development that warrant further investigation.

In a major series involving IgG4-TIN patients, 90% with elevated sCr levels at presentation, once treated with steroids, exhibited decreased sCr levels at follow-up, IgG4-TIN has a sensitive response to hormone, similar to other organ manifestations of IgG4-RD.^[23,31]^ A small case report described a response to rituximab in IgG4-RD patients refractory to steroid treatment.^[32]^ In a series of IgG4-MN patients, 7 were treated with various drugs, 2 of whom exhibited a rapid response only with corticosteroid treatment. In view of our case, corticosteroid therapy was useful in IgG4-related membranous glomerulonephritis. As for MGUS, treatment is not recommended until progression to multiple myeloma, and appropriate monitoring of patient is essential.^[33]^

## Author contributions

**Clinicopathologic analysis:** Jing Yi Sun.

**Conceptualization:** Xiao Ying Ma, Hai Ping Xu, Fu Yun Sun.

**Data curation:** Xiao Ying Ma.

**Formal analysis:** Xiao Ying Ma.

**Funding acquisition:** Fu Yun Sun.

**Investigation:** Xiao Ying Ma, Fu Yun Sun.

**Methodology:** JingYi Sun.

**Project administration:** Fu Yun Sun.

**Resources:** Fu Yun Sun.

**Software:** Xiao Ying Ma.

**Supervision:** Xiao Ying Ma, Fu Yun Sun.

**Validation:** Fu Yun Sun.

**Visualization:** Fu Yun Sun.

**Writing – original draft:** Xiao Ying Ma.

**Writing – review & editing:** Xiao Ying Ma, Yuresha Surangani Siyabalagaba Gedara.
